# Next-generation sequencing of Tunisian Leigh syndrome patients reveals novel variations: impact for diagnosis and treatment

**DOI:** 10.1042/BSR20220194

**Published:** 2022-09-21

**Authors:** Meriem Hechmi, Majida Charif, Ichraf Kraoua, Meriem Fassatoui, Hamza Dallali, Valerie Desquiret-Dumas, Céline Bris, David Goudenège, Cyrine Drissi, Saïd Galaï, Slah Ouerhani, Vincent Procaccio, Patrizia Amati-Bonneau, Sonia Abdelhak, Ilhem Ben Youssef-Turki, Guy Lenaers, Rym Kefi

**Affiliations:** 1 Laboratory of Biomedical Genomics and Oncogenetics, Institut Pasteur de Tunis, BP 74, 13 Place Pasteur, Tunis 1002, Tunisia; 2 National Institute of Applied Science and Technology, University of Carthage, Tunis, Tunisia; 3 University of Tunis El Manar, 2092 El Manar I Tunis, Tunisia; 4 University of Angers, MitoLab Team, Unité MitoVasc, UMR CNRS 6015, INSERM U1083, SFR ICAT, Angers, France; 5 Genetics and Immuno-Cell Therapy Team, Mohammed First University, Oujda, Morocco; 6 Research Laboratory LR18SP04, Department of Child and Adolescent Neurology, National Institute Mongi Ben Hmida of Neurology, Tunis, Tunisia; 7 Department of Biochemistry and Genetics, University Hospital Angers, Angers, France; 8 Department of Neuroradiology, National Institute Mongi Ben Hmida of Neurology, Tunis, Tunisia; 9 Department of Clinical Biology, Research Laboratory LR18SP04, Department of Child and Adolescent Neurology, National Institute Mongi Ben Hmida of Neurology, Tunis, Tunisia; 10 Laboratory of Proteins Engineering and Bioactive Molecules (LIP-MB), National Institute of Applied Sciences and Technology of Tunis (INSAT), The University of Carthage, Tunis, Tunisia

**Keywords:** Leigh syndrome, mitochondrial cytopathies, NGS, North Africa, Tunisia

## Abstract

Mitochondrial cytopathies, among which the Leigh syndrome (LS), are caused by variants either in the mitochondrial or the nuclear genome, affecting the oxidative phosphorylation process. The aim of the present study consisted in defining the molecular diagnosis of a group of Tunisian patients with LS.

Six children, belonging to five Tunisian families, with clinical and imaging presentations suggestive of LS were recruited. Whole mitochondrial DNA and targeted next-generation sequencing of a panel of 281 nuclear genes involved in mitochondrial physiology were performed. Bioinformatic analyses were achieved in order to identify deleterious variations.

A single m.10197G>A (p.Ala47Thr) variant was found in the mitochondrial *MT-ND3* gene in one patient, while the others were related to autosomal homozygous variants: two c.1412delA (p.Gln471ArgfsTer42) and c.1264A>G (p.Thr422Ala) in *SLC19A3*, one c.454C>G (p.Pro152Ala) in *SLC25A19* and one c.122G>A (p.Gly41Asp) in *ETHE1*.

Our findings demonstrate the usefulness of genomic investigations to improve LS diagnosis in consanguineous populations and further allow for treating the patients harboring variants in *SLC19A3* and *SLC25A19* that contribute to thiamine transport, by thiamine and biotin supplementation. Considering the Tunisian genetic background, the newly identified variants could be screened in patients with similar clinical presentation in related populations.

## Introduction

Mitochondrial diseases (MD) are multisystemic, encompassing a wide phenotypic spectrum, and can be manifested at any age [1,2]. MD affect approximately 1 in 5000 and are characterized by the alteration of the mitochondrial oxidative phosphorylation system (OXPHOS), composed of five complexes embedded in the mitochondrial inner membrane [3]. They are caused either by variants in the mitochondrial DNA (mtDNA) and nuclear DNA (nDNA), with >300 nuclear genes involved in the composition of the OXPHOS complexes, in the biogenesis and structure of the mitochondria, the mtDNA maintenance and in metabolic pathways [4,5].

Leigh syndrome (LS), also known as subacute necrotizing encephalo-myelopathy, was first described by Archibald Leigh in 1951, as a progressive psychomotor retardation or regression, induced by a symmetrical necrosis in the brain stem, thalamus and basal ganglia. Other LS-associated symptoms are hypotonia, muscle weakness, cerebellar ataxia, spasticity, nystagmus, optic atrophy, dysarthria, failure to thrive due to dysphagia, and elevated lactate levels in blood and in the cerebrospinal fluids. The disease usually appears in the first year of life and death may occur after respiratory muscle failure [6,7].

LS inheritance is complex since it involves maternal, autosomal recessive and dominant, and X-link modes of transmission. MtDNA variations are found in approximately 20% of cases, affecting mainly the respiratory complexes I, IV and V, and among them, the m.8993T>G in *MT-ATP6* is the most frequent, altering the complex V efficiency. The other 80% of LS cases involve variations in nuclear genes, altering essentially the function or assembly of the OXPHOS complexes, mostly resulting in complex I deficiency [8,9].

In Tunisia, several genetic studies on LS patients were performed leading to the identification of variants in both mtDNA and nDNA. For example, the m.8993T>G variant in *MT-ATP6* was detected with variable heteroplasmic loads in all affected members of a single family [10]. Two novel mitochondrial variants, m.5523T>G and m.5559A>G located in *MT-TW* affecting conserved regions of the *tRNA^trp^*, were also identified [11] as well as the m.9478T>C missense variant (V91A) in *COXIII* [12]. Alternatively, variants in *SURF1* were identified, either as a homozygous splice site c.516-517delAG or as compound heterozygous c.752-18A>C plus c.751+16G>A, most probably altering *SURF1* mRNA splicing and consequently the abundance of SURF1 protein [13].

The aim of this study was to further characterize genetic alterations in the mitochondrial and nuclear genomes in a cohort of Tunisian LS patients.

## Patients and methods

### Patient’s descriptions

**Figure 1:**
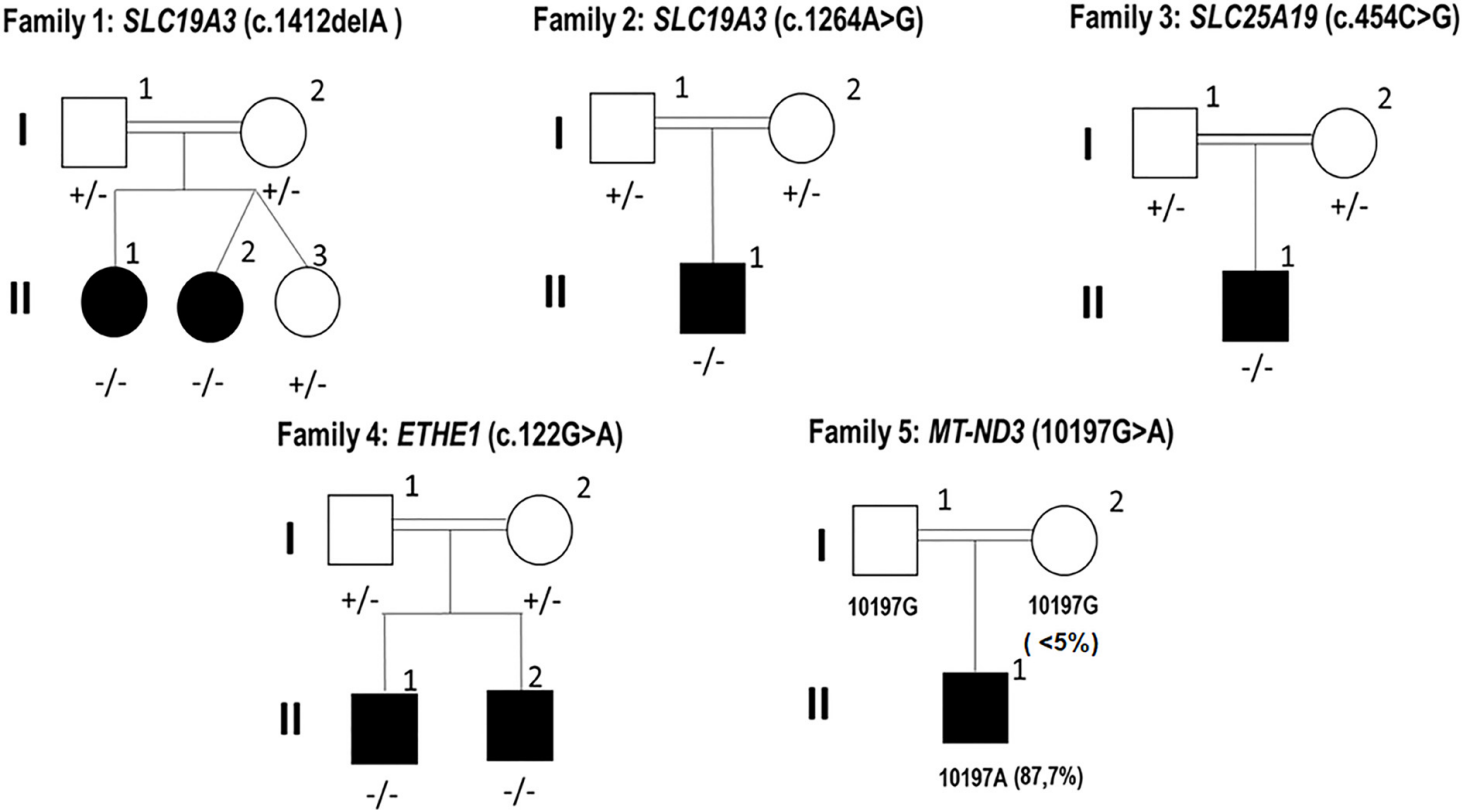
Pedigrees of the investigated families and segregation of their pathogenic variants Double lines indicates consanguineous parents; +/- indicates heterozygous for the variant; +/+ homozygous for the normal allele and -/- homozygous for the deleterious variant. The position of each variation is in-between brackets.

The present study was conducted according to the declaration of Helsinki and following the IRB recommendations and was approved by the Ethical Committee of The Institut Pasteur de Tunis Tunisia (Registration number IRB00005445, FWA00010074, Reference 2017/28/ILR16IPT). Written informed consent was obtained from parents for all the patients under the age of 18 years. Blood samples were obtained from five Tunisian families with six children having clinical, radiological and biochemical data suggestive of LS (Figure 1). They were recruited from the Department of Pediatric Neurology (National Institute Mongi Ben Hmida of Neurology, Tunis).

### Family 1 patient 1

An 8-year-old girl born from first-degree consanguineous parents. She had no perinatal asphyxia and a normal psychomotor development. Walking unaided was acquired at the age of 18 months. She was admitted to the department of pediatric neurology at the age of 5 years for acute gait disorder, dysarthria and abnormal posture. Examination revealed a right hemiparesis and generalized dystonia with dystonic dysarthria. Brain magnetic resonance imaging (MRI) revealed a bilateral necrosis of striatum, T2 weighted hyperintensity of the midbrain, with subcortical white matter and mammillary tubercles. The spectroscopy showed doublets of lactate. She had high levels of lactate in blood (2.82 mmol/l; normal values 0.5–2.2 mmol/l) but normal in the cerebrospinal fluid (1.38 mmol/l; normal values <2 mmol/l). The lactate and pyruvate analysis revealed an elevated lactate-to-pyruvate ratio of 36.78, with a plasma lactate levels 2.06 mmol/l and pyruvate concentration of 0.056 mmol/l. This patient does not have other disorder than this diagnosis and no family history for LS.

### Family 1 patient 2

She is a 6-year-old girl born from a twin pregnancy. She is the younger sister of patient 1. Her dizygotic twin sister is healthy. She had no perinatal suffering and normal psychomotor development. The onset of the disease began at the age of 3 years and 8 months with acute abdominal pain followed by a gait instability, bilateral divergent strabismus and drowsiness without fever. Clinical examination revealed a drowsy patient spastic tetraparesis, an unsteady gait, axial hypotonia, pyramidal signs in four limbs, generalized dystonia, bilateral nystagmus, divergent strabismus and ptosis. Computerized Tomography scan (CT-scan) showed a hypodensity of caudate nuclei and thalami. The MRI revealed T2 and FLAIR weighted hyperintensity of thalami, striatum, vermis, peri-aqueducal region and fronto-temporo-parietal cortex without enhancement and without restriction of diffusion. She had no lactate peak on spectroscopy. Her CSF lactate levels were normal (1.4 mmol/l). The lactate and pyruvate ratio appeared to be normal (18.16), with a plasma lactate level of 2.18 mmol/l and pyruvate concentration of 0.12 mmol/l. No other genetic disorder is diagnosed for this patient.

### Family 2 patient 1

He is a 7 years old boy born from first-degree consanguineous parents. He had a personal history of perinatal asphyxia. He had a normal psychomotor development. He presented at the age of 14 months abnormal posture of upper limbs and gait disorders that rapidly worsened until a complete loss of the ability to walk. He had concomitant flu syndrome. Clinical examination showed that the patient was apyretic, drowsy and irritable. He had quadri-pyramidal syndrome and generalized dystonia. The first brain MRI performed at 7 days of evolution and showed abnormalities in subcortical white matter and in basal ganglia. The diagnosis of acute disseminated encephalomyelopathy (ADEM) was evoked initially. He was hospitalized in the department of pediatric neurology. A second brain and spinal MRI was performed and revealed T2 and FLAIR weighted hyperintensity in basal ganglia with necrosis of the central area of the striatum suggestive of LS. Spectroscopy showed a lactate peak. CSF lactate was 1.47 mmol/l. Electromyoneurography (EMNG) was in favor of axonal sensory neuropathy of the lower limbs. He was treated as ADEM with corticosteroids and thiamin. The evolution was marked by improvement of consciousness and contact. However, he kept a severe generalized dystonia. The MRI performed 4 years later and showed a striatum atrophy with persistent hyperintensity. The lactate-to-pyruvate ratio was normal (17.2), with lactate plasma levels 1.38 mmol/l and pyruvate concentration 0.08 mmol/l. No other genetic disease is found and no family history for LS for this patient.

### Family 3 patient 1

He is a 4-year-old boy born from first-degree consanguineous parents. As for family history he had three aunts that died for an unknown reason between the ages of 4 and 5 years old. He had initially a normal psychomotor development. Walking was acquired at 14 months. He had a language delay. At the age of 14 months, he was hospitalized with 39°C fever, generalized epileptic seizures, sleep disturbance and psychomotor regression with loss of head control, sitting station, standing and walking. Examination showed generalized hypotonia and irritability. The diagnosis of encephalitis was evoked initially and the patient was treated by acyclovir and sodium valproate. One month later, he presented dyspnea and dehydration. Biochemical analysis showed cytolysis. Pyruvate blood levels before and after meal were 0.145 and 0.217 mmol/L, respectively. Brain MRI showed thrombosis of the left lateral sinus, and bilateral T2 and FLAIR weighted hyperintensity of striatum were observed. MRI spectroscopy showed a peak of lactate. He was switched to phenobarbital and received heparinotherapy during 3 months. The evolution was marked by improvement of motor skills. However, he presented recurrent episodes of drowsiness. Brain MRI performed 3 years later showed striatum necrosis with peak of lactate suggestive of LS. The lactate-to-pyruvate ratio was elevated 40.12, with lactate plasma levels 5.34 mmol/l and pyruvate levels 0.133 mmol/l. No other genetic disorder is diagnosed for this patient.

### Family 4 patient 1

A 5-year-old boy born from second-degree consanguineous parents. He had a brother with similar clinical signs who died at the age of 7 years. He was admitted to the hospital at the age of 5 months with chronic glairy diarrhea without fever. Examination showed axial hypotonia associated with spastic tetraparesis and generalized dystonia. He started walking at the age of 2 years and 2 months and his language skills were at monosyllable stage at the age of 2 years. MRI revealed lacunar lesion in the putamen and the anterior limb of the internal capsule. CSF lactate level was high (3.13 mmol/l). The lactate-to-pyruvate ratio was elevated 23.06, with lactate plasma levels 3.69 mmol/l and pyruvate levels 0.16 mmol/l. This patient does not have other disorder than this diagnosis.

### Family 5 patient 1

A 5-year-old boy born from third-degree consanguineous parents. As for family history he had a 7-year-old maternal cousin who had diabetes and developed psychomotor regression at 5 years old. Pregnancy and delivery were uneventful. Psychomotor development was normal in the first year. His language acquisition was at babbling stage. The disease started at the age of one year with a motor regression followed by a complete loss of ambulation. He was unable to sit or stand independently. He was hospitalized at 15 months of age for investigation. Examination showed axial hypotonia, quadripyramidal syndrome and dystonia of the upper limbs. Brain MRI showed a T2 and FLAIR weighted hyperintensity of basal ganglia of the periaqueductal region and the *substantia nigra* with a decrease in apparent diffusion coefficient (ADC). Spectroscopy revealed a doublet of lactate. Visual evoked potential, brain stem evoked response, cardiac and abdominal ultrasound were normal. EMNG was myogenic. CSF lactate levels were elevated (3.30 mmol/l). Evolution was marked by improvement of motor skills with appearance of generalized dystonia. He also developed epilepsy with tonic seizures treated by lamotrigine with improvement. This patient does not have other disorder than this diagnosis.

### Methods

#### Targeted next-generation sequencing

Total DNA was extracted from blood using the FlexiGene DNA kit (Qiagen). DNA quality was assessed using nano-drop spectrophotometer (Thermofisher scientific) and Qubit 2.0 (ThermoFisher scientific). As previously described by [14], whole mitochondrial genome was amplified using two 8.5 kilo base (kb) over-lapping fragments. Ion Plus Fragment Library kit (Cat.no.4471269) was used in order to prepare the mtDNA library. mtDNA rearrangement and deletions were establish using eKLIPse software [15].

A customized NGS panel of 281 nuclear genes involved in the maintenance of the mitochondrial genome, the assembly and function of the OXPHOS complexes, and the mitochondrial biogenesis was used to screen pathogenic variants for the six patients and their parents. Library preparation, sequencing and bioinformatics analysis were processed as described elsewhere [14].

#### Confirmation of variants by Sanger sequencing

Sanger sequencing was performed to confirm all the identified variants and to test the family segregation. Exons with candidate variants were amplified by PCR and sequenced on an ABI PRISM 3100- Avant automated DNA sequence using the BigDye Terminator Cycle sequencing reaction kit v1.1. Sequences were compared with the updated Cambridge sequence of the mtDNA (GenBank accession number: NC_012920). Sequence analyses were performed using SeqScape.

## Results

### Mitochondrial DNA screening for variants and deletions

The mitochondrial genome from blood was screened for the six index patients. Results revealed only in the index case of family 5, the presence of m.10197G>A (p.Ala47Thr) variant in *MT-ND3* with a heteroplasmic rate of 87.7%. This variant is considered as probably damaging and deleterious according to PolyPhen and Combined Annotation-Dependent Depletion (CADD), respectively. This variant has previously been reported with complex I deficiency disorders but newly identified for the Tunisian population in our patient with LS. The data of maternal heteroplasmy are <5%. Indeed, we performed whole mitochondrial DNA sequencing for the mother we did not find the mutation. We checked for the coverage of the gene region and it was sufficiently covered. We performed Sanger sequencing, we found that the mother was wild-type for the mutation. Consequently, the heteroplasmy rate for the mutation is <5% since it cannot be detected in Sanger sequencing performed on DNA extracted from blood.

Analysis of mtDNA sequences of all patients using eKLIPse software did not reveal rearrangement or deletion (data not shown).

### Screening nuclear DNA for variations

Next generation sequencing of a panel of 281 genes involved in mitochondrial physiology was performed for the five patients devoid of mtDNA variant. Using a filtering step [14], four pathogenic variations in *SLC19A3*, *SLC25A19* and *ETHE1* genes (Table 1) were identified.

**Table 1:** Variants identified in Tunisian patients with LS

Family and Patients	G	Age	Gene	ORF variant	Position	Protein change	PolyPhen	Sift	Mutation Taster	LRT	Mutation Assessor	MetaRNN	rs number
Family 1													
Patient 1	F	6	SLC19A3	NM_025243.4: c.1412delA	Chr2: 228552192	p.Gln471ArgfsTer42	–	–	–	–	–	–	–
Patient 2	F	5	SLC19A3	NM_025243.4: c.1412delA	Chr2: 228552192	p.Gln471ArgfsTer42	–	–	–	–	–	–	–
Family 2													
Patient 1	M	5	SLC19A3	NM_025243: c.1264A>G	Chr2: 228552932	p.Thr422Ala	Damaging	Damaging	Disease causing	Deleterious	High	Damaging	rs121917884
Family 3													
Patient 1	M	4	SLC25A19	NM_001126122.1: c.454C>G	Chr17: 73279509	p.Pro152Ala	Possibly damaging	Damaging	Disease causing	Deleterious	Medium	Damaging	–
Family 4													
Patient 1	M	3	ETHE1	NM_014297: c.122G>A	Chr19: 44030771	p.Gly41Asp	Probably damaging	Damaging	Disease causing	Deleterious	Medium	Damaging	–
Family 5													
Patient 1	M	3	MT-ND3	m. 10197G>A	mitochondria	p.Ala47Thr	–	–	–	–	–	–	–

G: gender; M: male; F: female.

Both sisters from family 1 (patient 1 and 2) harbored the same homozygous frameshift deletion c.1412delA in *SLC19A3* exon 6. This novel variant was heterozygous in both parents and in the dizygotic twin sister that leads to a frameshift p.Gln471ArgfsTer42 classified as likely pathogenic = PP1/PM2/PM4 according to the ACMG classification (the American college of medical genetics). A second variation c.1264A>G in *SLC19A3* exon 5 was found in family 2 patient 1. Both parents were heterozygous for this variant. This variation is predicted to be pathogenic = PS1/PS3/PP1/PM2/PM3 according to the ACMG classification and was linked to biotin-thiamine responsive basal ganglia disease (BTRBGD) as a founder mutation for the Saudi Arabian population. And previous functional studies support our findings by demonstrating that this variant down-regulate the thiamine transport activity of THTR2.

Family 3 patient 1 carried a novel homozygous missense c.454C>G variation in *SLC25A19* exon 5, responsible for proline to alanine change in position 152. This variant was predicted to be deleterious according to Sift, PolyPhen and Mutation Taster. This variant was also classified according to the ACMG classification criteria as likely pathogenic (PM2, PM5 and PP3). No functional studies for this variant have been reported to support the likely pathogenic effect which still present some ambiguity about this impact for this variant, which is recurrently the case with novel variants in rare disease genes, so further functional studies need to be performed to showcase the impact of this variant. Both parents were heterozygous for this variant.

Finally, a novel homozygous missense variation c.122G>A variant in *ETHE1* was identified in patient 1 from family 4. This caused an amino acid substitution of glycine to aspartate at the protein position 41 (p.Gly41Asp). Sanger sequencing revealed that both parents were heterozygous for this variation, which is predicted to be damaging and disease causing by PolyPhen, Sift and Mutation Taster. And according to the ACMG classification criteria it was predicted as uncertain significance (PM2 and PP2). Biochemical investigation of the respiratory complexes’ activities and cellular respiration of a patient biopsy showed normal values (data not shown).

## Discussion

### Impact of NGS/TGS on the diagnosis of LS patients

In the present study, we report five consanguineous Tunisian families with six children affected by LS. Mitochondrial genome sequencing revealed a novel m.10197G>A variation in family 5, with high heteroplasmic rate (87.7%), which has never been reported before in Tunisia, but well in patients affected by encephalo-myopathy [16], Leigh like syndrome [17], mitochondrial diseases with complex I deficiency [18], progressive generalized dystonia of childhood, Leber hereditary optic neuropathy with dystonia [19] and in another patient with stroke like episodes [20]. In addition, three patients with LS or MELAS/LS overlapping syndromes harbored this m.10197G>A variant. High heteroplasmic rates of this variant can alter complex 1 activity and decrease cell respiration by 30% [21,22], and may explain the clinical features of our patient.

NGS sequencing of 281 genes implicated in mitochondrial diseases identified four variations, among them three novels in three different genes. These novel variants were checked in 100 unrelated Tunisian controls and were not found. The first variation c.1412delA (p.Gln471ArgfsTer42) in *SLC19A3* in the two sisters of family 1 was homozygous and led to a highly similar clinical phenotype. This variant created a new nonsense variant further downstream destroying the initial stop codon. This variant will lead to an abnormal protein with longer peptide chain altering the 3D structure and subsequently its function. Added to that since the alteration occurred on the 3’ untranslated region it will alter the regulatory function of this region [23]. Although further functional studies need to be performed to assess the pathogenicity of this variant and its impact on the protein expression and the patho-mechanism associated with LS. The variant c.1264A>G in *SLC19A3* found in family 2 results in a Threonine to Alanine change at position 422, altering a transmembrane domain. It was already reported in three families originated from Saudi Arabia [24] leading to decreased thiamine transporter 2 (hTHTR2) activity and down-regulation of biotin accumulation [25]. Furthermore, this c.1264A>G variation was identified as a founder variant in the Saudi Arabian population for BTRBGD [26].

The *SLC19A3* gene encodes for hTHTR2, an ubiquitously expressed thiamine transporter, composed of 12 transmembrane domains [25]. Additional *SLC19A3* variations were described in Leigh and Leigh-like syndromes [27,28], in BTRBGD disease [29,30] and in Wernicke’s encephalopathy [31]. To date there is a total of 62 disease-causing variants identified in the *SLC19A3* gene with our findings, which are associated with a wide spectrum of disorders from mild-to-severe clinical manifestations. Among these variants 37 are missense, 18 truncating mutations (nonsense or frameshift), four gross deletions of either exons, promoter region or the whole gene and 2 splicing mutations [32]. A novel homozygous missense variant c.958G>C (p.Glu320Gln) was found in four Japanese patients with a clinical presentation and radiological traits that were classified as LS. The p.Glu320Gln variant decreased of 63% the thiamine uptake [33]. Considering a case with adult-onset Wernicke’s-like encephalopathy a compound heterozygous variants Glu320 Gln combined with Lys44Glu were identified leading to a mild clinical manifestation [31]. Other than that, the hot spot variant identified Thr422Ala in the Saudi Arabian result in a somewhat mild phenotypes with a good prognosis [32,34]. As for the truncated variants, a Turkish patient harbored the *SLC19A3* homozygous frameshift variant c.982del (p.Ala328Leufs*10), resulting in a truncated protein. This loss-of-function mutation reported in a case of early onset Leigh like syndrome and rapidly fatal prognosis [27]. The *SLC19A3* homozygous c.20C>A variant causing a premature stop codon was found in two Moroccan siblings with LS, and then in 17 other LS patients, nine of these patients soon died after diagnosis [35]. Pyruvate dehydrogenase activity from a muscle biopsy of one LS patient was virtually null, but the addition of thiamine pyrophosphate (TPP) did restore this activity [35]. Still genotype–phenotype remains unclear but usually homozygous loss-of-function variants are associated with more severe phenotypes with infantile disease onset and a poor vital prognosis compared with missense variants.

The second thiamine transporter *SLC25A19* gene was found mutated in family 3, disclosing for the first time the c.454C>G (p.Pro152Ala) variant in a Tunisian patient. Alterations of *SLC25A19* gene have been linked to the bilateral striatal necrosis and neuropathy characterized by a truncal ataxia and hypotonia, episodes of encephalopathy, swallowing problems and recurrent episodes of flaccid paralysis and progressive polyneuropathy [36] and with the Amish lethal microencephalopathy. This latter disease is characterized by an apparent congenital microcephaly, high levels of α-ketoglutarate in urine, brain malformations and psychomotor retardation with encephalopathy episodes [37]. Li et al. identified the same variant c.454C>A p.(Pro152Thr) but not the same amino acid change, in a Chinese LS patient, as compound heterozygous together with another variation c.194C>T (p.Ala65Val) in the same gene [38]. Variants of *SLC25A19* play a major role on the etiology of LS, since neurological LS symptoms are convergent toward a patho-mechanism of diseases related to thiamine transport. The members of thiamine transporters are expressed ubiquitously and thiamine plays a major role in the proper maintenance of the nervous, cardiovascular and motor systems [39,40]. But still, further functional studies need to be conducted in order to show beyond doubt the impact of this variant on LS pathophysiology since it is a bit ambiguous to demonstrate the effect for novel variant on their classification as disease causing.

Family 4 patient 1 presented a novel *ETHE1* homozygous variant c.122G>A,p.(Gly41Asp). *ETHE1* variants have been previously described as responsible for the development of ethylmalonic encephalopathy (EE), which is characterized by chronic diarrhea, recurrent petechiae, neurological degeneration, psychomotor delay, hypotonia, spastic tetraplegia, orthostatic acrocyanosis and Leigh-like syndrome [41,42]. EE was mistakenly diagnosed as a LS, since both presentations have common clinical and radiological features, such as psychomotor regression, brain lesions, pyramidal and quadri-pyramidal syndromes, hypotonia and ataxia. According to the literature one patient was diagnosed as LS at the beginning, then after the apparition of petechia, orthostatic acrocyanosis, mild hepatomegaly and high ethylmalonate in blood and urine levels, the diagnosis was reconsidered to EE. Importantly, enzymatic activities of the mitochondrial respiration complexes from EE patient fibroblasts were normal [43], as we found in the case of family 4.

Since all of our patients are born from consanguineous parents and the Tunisian population is known for a higher rate of consanguinity suggesting that autosomal recessive inheritance is a predominant mode of inheritance (62.9% of disease are autosomal recessive in the Tunisian population) [44], it would be more appropriate to screen the nuclear genome first and then if it is required the mitochondrial genome.

### Founder mutations in LS

With respect to founder effects in LS, previous studies have identified the *SLC19A3* c.20C>A variation in the Northern region of Morocco, in three unrelated families originated from the Al Houceima province [35]. Other founder variants were reported for LS patients in the literature. Variants with founder effect in *NDUFS4* gene was described in the Moroccan, Algerian and Ashkenazi Jewish population [45]. Homozygous variant in *PET100* was reported in Lebanese patients [46]. Variants in *LRPPRC* gene were found in patients originated from Northern Québec with LS French-Canadian type [47,48]. Variant in *NDUFS4* gene present in five patients with LS is suggestive of a founder effect for the Hutterite population [49]. A new founder variant in *USGM5* was identified for Ashkenazi Jewish patients affected with LS [50]. These LS founder variants were not found in our Tunisian LS patients. Nevertheless, the *SLC19A3* variation c.1264A>G rs121917884 observed in our patient (family 2 patient 1), was identified as a founder variant for the Saudi Arabian population for BTRBGD. These latter variants could be screened as founder variants for LS Tunisian patients.

### Strategies for treatments

In the context of LS, for which no efficient treatment is available in most cases, patients harboring variants in the thiamine transporter genes, like *SLC19A3* and *SLC25A19* can be treated by thiamine and biotin supplementation, in order to increase thiamine intra cellular and mitochondrial concentrations [25]. Thiamine enters the cytosol through the hTHTR2, then is converted to TPP, an active coenzyme, by the pyrophosphokinase 1 (TPK-1). Then, TPP is transported across the mitochondrial membrane by the thiamine pyrophosphate carrier encoded by *SLC25A19*. TPP stimulates the production of acetyl-COA through the conversion of pyruvate to fuel the Krebs cycle [39,51]. Thus, high doses of thiamine associated with biotin can be indicated as a treatment for LS patients with alteration of thiamine transporters in particular for those with variant in *SLC19A3* or *SLC25A19* [52]. Taking into consideration these recommendations, our Tunisian patients with *SLC19A3* and *SLC25A19* variants affecting thiamine transport were supplemented with high doses of thiamine: 250 mg/day and biotin 5–10 mg/kg/day. This led to a notable improvement and stabilization of the course of the disease, which was reached for patient 1 harboring the *SLC19A3* c.1412delA variant after only 11 days of treatment. As regards to patient 2 family 1, we observed a total recovery after treatment for 12 days. After 3 months of treatment, the patient 1 from family 2 has partially recovered and did not present any novel episodes of encephalopathy. Lastly, patient 1 from family 3 started the treatment late, we observed a gradual improvement by the acquisition of walking and talking despite another encephalitic episode.

Regarding patients with *ETHE1* variants, it was reported that they can be treated with metronidazole and neomycine in alternance, in addition to N-acetylcysteine in order to decrease H_2_S levels. Alternatively, liver transplant can also expand the life span of children with EE [53,54].

## Conclusion

The present study reports for the first time in Tunisia a NGS analysis of five families with children affected with LS. These patients were mutated in LS genes (*MT-ND3* and *SLC19A3*) and also in syndrome Leigh-like (*SLC25A19* and *ETHE1*). Taking into account the broad spectrum of identified variants in our cohort, patients with similar phenotype for Leigh or Leigh-like syndrome should be screed for the common variant previously identified for the sake of cost-effectiveness and to improve prognosis for treatable cases. And for patients without genetic diagnosis a wider genetic analysis should be performed such as whole mitochondrial sequencing, targeted gene sequencing and in some cases whole exome sequencing.

## Consent for Participation

All subjects gave written informed consent.

## Data Availability

Materials and raw data can be requested from the authors upon request.

## Abbreviations

ADC: apparent diffusion coefficient
BTRBGD: biotin-thiamine responsive basal ganglia disease
CADD: combined annotation-dependent depletion
EE: ethylmalonic encephalopathy
EMNG: electromyoneurography
LS: Leigh syndrome
MD: mitochondrial diseases
OXPHOS: oxidative phosphorylation system
TPP: thiamine pyrophosphate
